# Gelatin-Methacryloyl (GelMA) Formulated with Human Platelet Lysate Supports Mesenchymal Stem Cell Proliferation and Differentiation and Enhances the Hydrogel’s Mechanical Properties

**DOI:** 10.3390/bioengineering6030076

**Published:** 2019-08-28

**Authors:** Marline Kirsch, Luise Birnstein, Iliyana Pepelanova, Wiebke Handke, Jessica Rach, Axel Seltsam, Thomas Scheper, Antonina Lavrentieva

**Affiliations:** 1 Institute of Technical Chemistry, Gottfried Wilhelm Leibniz Universität Hannover, 30167 Hannover, Germany; 2 German Red Cross Blood Service NSTOB, 31832 Springe, Germany

**Keywords:** gelatin-methacryloyl (GelMA), hydrogels, 3D cell culture, human platelet lysate, adipose tissue-derived mesenchymal stem cells (AD-MSCs)

## Abstract

Three-dimensional (3D) cell culture is a major focus of current research, since cultivation under physiological conditions provides more reliable information about in vivo cell behavior. 3D cell cultures are used in basic research to better understand intercellular and cell-matrix interactions. Moreover, 3D cell culture plays an increasingly important role in the in vitro testing of bioactive substances and tissue engineering. Gelatin-methacryloyl (GelMA) hydrogels of different degrees of functionalization (DoFs) are a versatile tool for 3D cell culture and related applications such as bioprinting. Human platelet lysate (hPL) has already demonstrated positive effects on 2D cell cultures of different cell types and has proven a valuable alternative to fetal calf serum (FCS). Traditionally, all hydrogels are formulated using buffers. In this study, we supplemented GelMA hydrogels of different DoF with hPL during adipose tissue-derived mesenchymal stem cell (AD-MSCs) encapsulation. We studied the effect of hPL supplementation on the spreading, proliferation, and osteogenic differentiation of AD-MSCs. In addition, the influence of hPL on hydrogel properties was also investigated. We demonstrate that the addition of hPL enhanced AD-MSC spreading, proliferation, and osteogenic differentiation in a concentration-dependent manner. Moreover, the addition of hPL also increased GelMA viscosity and stiffness.

## 1. Introduction

Three-dimensional (3D) cell culture systems provide a more physiological environment for cells in comparison to traditional 2D cell cultures in terms of cell-cell and cell-matrix interactions and diffusion behavior. Indeed, in 2D cell cultures, signaling molecules released by cells are immediately diluted in the greater volume of the cell culture medium instead of acting in a concentration- and site-dependent manner on other cells. Moreover, no physiological gradients of signaling molecules, metabolites, and oxygen can be created in 2D culture systems. For these reasons, 3D cultures are now increasingly used in basic research, drug screening, toxicity studies, and tissue engineering (TE) [1,2,3,4]. There are several 3D cell culture models that are used for these applications: scaffold-free cellular aggregates, cells growing on natural or synthetic scaffolds, and cells encapsulated in hydrogels [5,6]. The latter have become highly popular, since hydrogels can be made of a great variety of synthetic and natural molecules, and their properties can be adjusted according to cell model requirements [7,8,9]. Among others, gelatin-methacryloyl (GelMA) provides a broad spectrum of tunable parameters, which lead to the creation of hydrogel networks with different stiffness and pore architectures [10,11,12,13]. Thus, these hydrogels can serve as a versatile platform for 3D cell culture research and TE. Mesenchymal stem cells (MSCs) are widely used in biomedical applications. These include the use of MSCs in cell therapies for different indications and as part of TE constructs. Although cell therapies using MSCs are currently involved in over 900 clinical trials, the field of tissue engineering is still under development [14]. One of the challenges in this case is the development of suitable scaffolds. In the case of soft tissues, decellularized human tissues could be used as a TE scaffold. This strategy, however, has several limitations, such as possible rejection in case of allogenic transplantation [15,16]. Thus, engineered polymers like hydrogels represent a valuable alternative to decellularized tissues. 

On the one hand, the cultivation of cells in 3D systems recapitulates the in vivo microenvironment and diffusion rates in a better manner. On the other hand, slower diffusion rates can slow down cell adhesion and nutrient supply. Indeed, in comparison to 2D cell cultures, cell adhesion in hydrogels is much slower [17]. One reason for this observation could be that cell-containing constructs are traditionally formulated from hydrogels reconstituted in buffers (e.g., in phosphate buffer saline (PBS)). Even if the hydrogels in question possess enough arginine-glycine-aspartic acid (RGD) motifs for focal adhesion, typical adhesion proteins like fibronectin are still missing inside the bulk hydrogel and need to diffuse from the media into the hydrogel to exert their effects. 

Existing studies have shown that human platelet lysate (hPL) provides cells cultivated in vitro with numerous growth factors and small molecules required for growth and proliferation [18,19,20,21,22,23,24,25,26,27,28]. hPL increases cell proliferation and differentiation in 2D and can be used as a xeno-free supplement for various types of primary cells, including MSCs [18,19,22,23,24,25,26,27,28,29,30]. hPL can be easily produced from human platelet concentrate in conformity with good manufacturing practice (GMP) guidelines [28]. Thus, hPL appears to be a valuable alternative to FCS. Moreover, the application of hPL complies with modern social principles of ethics, sustainability, recycling, and conservation of resources. Platelets are donated worldwide within the scope of blood donation campaigns, though more than twenty percent of donated platelets expire before they can be used for infusions, due to their short shelf life. Since no differences were detected between the use of hPL prepared from expired or fresh platelet concentrates as a medium supplement, it is possible and desirable to recycle expired platelets obtained from blood banks [31]. 

In the present study, we hypothesized that the formulation of GelMA hydrogels with hPL will be beneficial for MSCs in terms of cell spreading, proliferation, and osteogenic differentiation. The effect of hPL addition on the mechanical properties of GelMA has also not been studied yet. In this work, we investigated (1) the influence of hPL on MSCs cultivated in hydrogels and (2) the influence of hPL on hydrogel viscosity and stiffness.

## 2. Materials and Methods 

### 2.1. GelMA Synthesis and Hydrogel Preparation 

GelMA of different degrees of functionalization (DoF) (50%, 70%, and 95%) was synthesized and analyzed as described earlier [10]. GelMA solutions were prepared at a concentration of 5% (w/v) by weighing an appropriate amount of GelMA and dissolving it in phosphate-buffered saline (PBS, pH 7.4), in PBS with the addition of 2.5%, 50% hPL (% v/v), or by 100% hPL (hPL pH value 7.3, manufactured by German Red Cross, Blood Service NSTOB, Springe, Germany). The GelMA solutions were incubated in a water bath until complete dissolution of the solid GelMA at 37 °C. All GelMA solutions contained 2-hydroxy-4′-(2-hydroxyethoxy)-2-methylpropiophenone (Irgacure 2959) at a final concentration of 0.1% (w/v). Warm GelMA solutions were sterile filtered prior to cell experiments using 0.45-μm polyethersulfone (PES) filters under aseptic conditions.

### 2.2. Rheological Characterization

GelMA viscosity was determined at 20 °C and 37 °C by rotational viscosimetry using an MCR 302 modular rheometer (Anton Paar, Graz, Austria) equipped with a plate-plate geometry (20-mm plate diameter) and a gap size of 1 mm. The shear rate was varied from 0.1–1000 s^−1^, which is a typical shear rate region associated with pneumonic extrusion bioprinting. 

The storage modulus of GelMA hydrogels with and without the addition of hPL was investigated in situ at 25 °C using an MCR 302 modular rheometer (Anton Paar, Graz, Austria) equipped with a plate-plate geometry (20-mm diameter, 1-mm gap size). The sample volume of 360 µL of GelMA with 0.1% (w/v) photoinitiator (PI) was filled in between the gap and was polymerized by performing in situ UV cross-linking by irradiation with a UV lamp (Delolux 80, Delo, Windach, Germany) from below, with a UV intensity of 20 mW/cm^2^ and UV irradiation time of 5 min. The storage modulus was recorded with a time sweep oscillatory test under a constant strain amplitude of 1% and at a constant frequency of 1 Hz, which is within the linear viscoelastic (LVE) region. The parent gelatin material was dissolved, and a sample volume of 400 µL was filled in silicon molds (20-mm diameter) and solidified for an hour at room temperature. The solid construct was placed in the rheometer, and the storage modulus was recorded with a time sweep oscillatory test.

### 2.3. Hydrogel Swelling

Polymerized GelMA hydrogels were incubated in water until equilibrium and weighed. The hydrogels were then frozen over night at −80 °C and lyophilized. The weight of the hydrogels after freeze-drying was also determined. The swelling ratio can be calculated from the weight after swelling (W_S_) and the weight of the dry hydrogel after lyophilization (W_D_) according to the following formula [32]:

$$\mathrm{Swelling}\;\mathrm{ratio}=\frac{\left(W_{S}-W_{D}\right)}{W_{D}}$$

### 2.4. Cell Culture

Human adipose tissue-derived mesenchymal stem cells (AD-MSCs) were isolated from adipose tissue after abdominoplasty. All patients provided their informed consent, as approved by the Institutional Review Board (Hannover Medical School) with the reference number 3475-2017. The isolated cell populations were characterized as mesenchymal stem cells by surface marker analysis, differentiation capacity, and functional properties [10]. Cells were expanded in alpha-MEM medium (Thermo Fisher Scientific, Waltham, MA, Germany) containing 1 g/L glucose, 2 mM·L-glutamine, 10% human serum (HS) (CC-pro, Oberdorla, Germany), and 50 μg/mL gentamicin (Merck Millipore, Darmstadt, Germany), harvested by accutase treatment (Merck KGaA, Darmstadt, Germany) and cryopreserved at Passage 1 until the start of the experiments. The experiments were performed with cells of Passages 2–8 only.

### 2.5. Encapsulation and Cultivation of AD-MSCs in Hydrogels

AD-MSCs were encapsulated in GelMA hydrogels at a concentration of 1.0 × 10^6^ cells/mL in 50-μL disks (6-mm diameter) in silicon molds with the help of a cross linker (BLX-365 Bio-Link, 365 nm, Vilber Lourmat, Germany)) with a UV intensity of 1.2 J/cm^2^ and a polymerization time of approximately 5 min. Polymerized hydrogel constructs with cells were incubated in 24-well plates in 400 μL of alpha-MEM medium (Thermo Fisher Scientific, Waltham, MA, Germany) containing 1 g/L glucose, 2 mM L-glutamine, 10% HS (CC-pro, Oberdorla, Germany), and 50 μg/mL gentamicin (Merck Millipore, Darmstadt, Germany). To avoid cell adhesion on the well bottoms, 1% agarose coating of wells was performed before cultivation. Hydrogel constructs with cells were cultivated in a humidified atmosphere containing 5% CO_2_ and 21% O_2_ at 37 °C. Indirect cell viability was estimated by the CellTiter-Blue^®^ (CTB) assay according to the manufacturer’s instructions (Promega, Mannheim, Germany). Briefly, CTB stock solution was added to the medium of the cells to receive a concentration of 10% CTB solution (1:10 v/v). After 5 h of incubation, fluorescence was measured at an extinction wavelength of 544 nm and an emission wavelength of 590 nm using a microplate reader (Fluoroskan Ascent, Thermo Fisher Scientific Inc., Waltham, MA, USA). Encapsulated AD-MSCs cultivated for one, three, or seven days were incubated in 4 µM calcein-acetoxymethyl (AM) (Merck, Darmstadt, Germany) solution in basal alpha-MEM for 40 min at 37 °C and analyzed with a Cytation 5-Cell Imaging Multi-Mode Reader (Biotek Instruments, Winooski, VT, USA).

### 2.6. Osteogenic Differentiation, Cryosections, and Alizarin Red Staining

AD-MSCs encapsulated in hydrogels with and without hPL were differentiated into osteoblasts. The osteogenic differentiation medium contained 5 mM β-glycerophosphate, 0.1 µM dexamethasone, 0.2 mM L-ascorbate-2-phosphate, 0.5% gentamicin, as well as 10% HS. The medium was exchanged every 3–4 days. Cells in hydrogels were cultured for 7, 14, or 21 days and then washed in PBS and fixed for 15 min at 4 °C with 4% paraformaldehyde (Merck, Darmstadt, Germany). Fixed hydrogels were embedded and frozen at −20 °C in Tissue-Tek^®^ O.C.T.™ Compound (Sakura Finetek Europe B.V., Alphen aan den Rijn, The Netherlands) for cryostat sectioning. Twelve-micrometer cryosections were made with the help of a Microm HM 560 cryostat (Thermo Fisher Scientific Inc., Waltham, MA, USA) at −20 °C and mounted on Super Frost glass slides (Thermo Fisher Scientific Inc., Waltham, MA, USA). For the evaluation of osteogenic differentiation, cryosections were incubated in Alizarin Red solution, containing 1% Alizarin Red S (Merck KGaA, Darmstadt, Germany) in deionized H_2_O, for 15 min at room temperature. After washing with deionized H_2_O, the red chelates were detected and analyzed with a fluorescent microscope (Olympus, IX50, Olympus Corporation, Tokyo, Japan), equipped with a camera (Olympus SC30, IX-TVAD, Olympus Corporation, Tokyo, Japan) and the CellSens Software (CellSens Standard 1.7.1, Olympus Corporation, Tokyo, Japan).

## 3. Results

### 3.1. AD-MSCs’ Viability in GelMA-Hydrogels

To evaluate the influence of hPL on 3D-cultivated AD-MSCs, cells were encapsulated in three different GelMA-hydrogels of 50%, 70%, and 95% DoF. Polymerization was performed at an intensity of 1.2 J/cm^2^, which has been shown to promote MSC spreading [10]. Each GelMA solution was prepared in PBS with the addition of 0%, 2.5%, or 50% hPL (% v/v) or by adding hPL only (100% hPL), prior to the addition of the photoinitiator and the polymerization of the hydrogels. The specific hPL concentrations used in this study were selected because: (1) 2.5% hPL is the recommended concentration for 2D cell cultivation [33,34]; (2) 100% represents a complete substitution of PBS with hPL; (3) 50% hPL serves as an intermediate point between 2.5% and 100% hPL; and (4) 0% hPL functions as a control. Calcein-AM staining was performed to evaluate the influence of hPL addition on cell viability and spreading. Cell proliferation in hydrogels was evaluated using a CTB assay.

**Figure 1:**
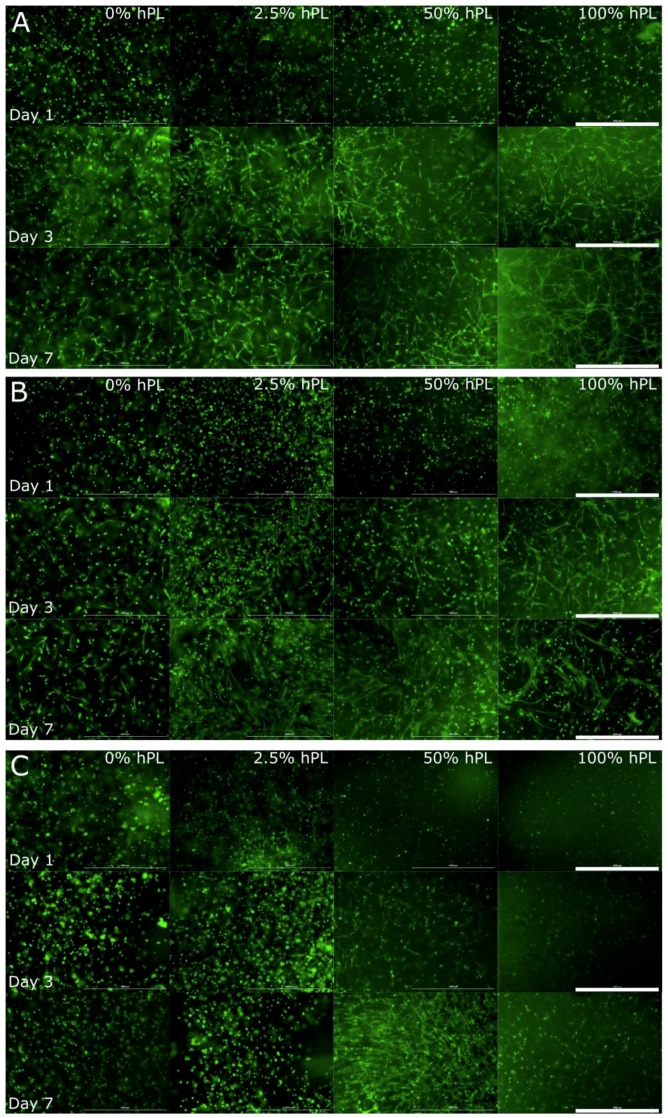
Morphological examination of AD-MSCs encapsulated with a UV dose of 1.2 J/cm^2^ in 5% GelMA with (**A**) 50% degree of functionalization (DoF), (**B**) 70% DoF, and (**C**) 95% DoF, formulated with 0, 2.5, 50, and 100% human platelet lysate (hPL). After cultivation of 1, 3, and 7 days, the cells were stained with calcein-AM; 4× objective, scale bar 1000 µm.

**Figure A1:**
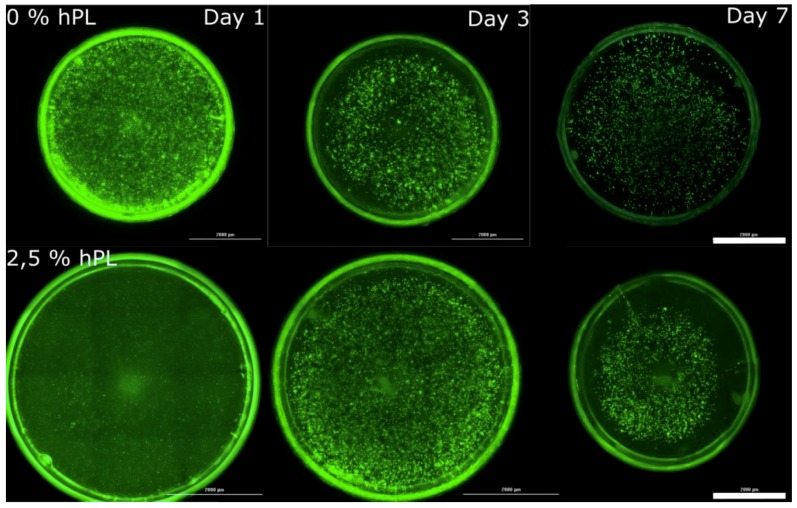
Cell aggregation of AD-MSCs in GelMA hydrogels (95% DoF) containing 0% and 2.5% hPL. Fluorescence pictures taken after one, three, and seven days of cultivation (4× objective and combined pictures). Scale bar 2000 µm per picture.

As shown in Figure 1, the calcein-AM staining indicated the presence of viable cells in all tested GelMA hydrogels. In comparison to cultivation in hPL-free materials, AD-MSCs demonstrated increased cell spreading and growth when cultivated in GelMA hydrogels containing hPL. Single cell spreading was detectable already one day after encapsulation of AD-MSCs in the lowest functionalized GelMA (50% DoF) formulated in hPL (100%) (Figure 1A). Cell spreading of AD-MSCs in GelMA hydrogels typically occurred after three days of cultivation [10]. In a similar manner, the AD-MSCs cultivated in hPL-free GelMA (hydrogels with 50% and 70% functionalization) showed only a few spreading cells after three days of cultivation (Figure 1A,B). A positive effect of adding hPL to GelMA could be seen by comparing the cell morphology in 0, 2.5, 50, and 100% hPL containing GelMA hydrogels on Day 7. The higher the hPL concentration, the more cells could spread and establish cell-cell contacts. AD-MSCs cultured in 100% hPL containing GelMA-hydrogels showed extensive cell-cell contacts and a pronounced three-dimensional network of cells. In contrast, AD-MSCs cultivated in GelMA hydrogels with a high DoF of 95% displayed a round morphology (Figure 1C). These cells exhibited no spreading or cell-cell contacts over the entire cultivation period. The only exceptions to this observation were cells cultivated in GelMA containing 50% hPL. Under these conditions, a few cells spread within the first 24 h and showed extensive cell spreading and three-dimensional cell growth after seven days of cultivation. Moreover, it was also noticeable that cell aggregates formed in hydrogels with 0% and 2.5% hPL after three and seven days, respectively. This is even more evident by comparing the images of the entire hydrogel (Figure A1). Since cells did not sufficiently spread and create cell-cell contacts in 95% DoF GelMA hydrogels, we excluded this material from further investigations of osteogenic differentiation.

**Figure 2:**
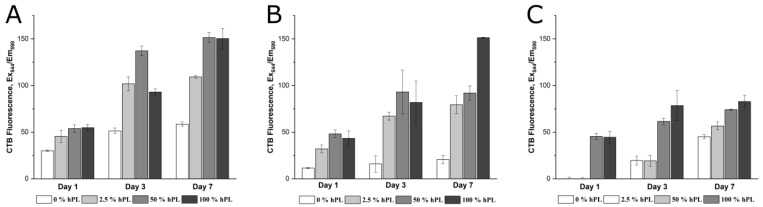
Cell viability of AD-MSCs encapsulated with a UV dose of 1.2 J/cm^2^ in 5% (w/v) GelMA with (**A**) 50% DoF, (**B**) 70% DoF, and (**C**) 95% DoF, dissolved in 0, 2.5, 50, and 100% hPL. The CellTiter-Blue^®^ (CTB) assay was performed on Day 1, Day 3, and Day 7 of cultivation. Data represent the mean ± SD for a threefold determination.

In addition to the calcein-AM staining, a CTB assay was performed to estimate the viability of the cells after one, three, and seven days of cultivation in GelMA hydrogels with 50%, 70%, and 95% of functionalization. As can be seen in Figure 2, a higher hPL concentration in the GelMA led to higher cell viability, at least on Day 7 of cultivation. Contrary to GelMA with DoF of 50% and 70%, cell viability in 95% DoF GelMA-hydrogels was increased until the third day by the addition of 50% and 100% hPL. These results suggest that platelet lysate contributes to the preservation and enhancement of cell viability. 

As shown before by Pepelanova et al., AD-MSCs cultivated in GelMA with a DoF of 70% or above were not able to spread and to establish cell-cell contacts [10]. The addition of hPL to GelMA, however, increased cell viability, supported cell spreading, and led to the formation of three-dimensional networks. The increased proliferation and viability can be attributed to growth factors contained in platelet lysate, such as epidermal growth factor (EGF), basic fibroblast growth factor (bFGF), platelet-derived growth factor (PDGF), and vascular endothelial growth factor (VEGF) [27]. Improved cell spreading after hPL addition can be explained by the influence of proteins like vitronectin and fibronectin, which mediate cell adhesion to the matrix [35].

### 3.2. AD-MSC Differentiation in GelMA-Hydrogels

**Figure 3:**
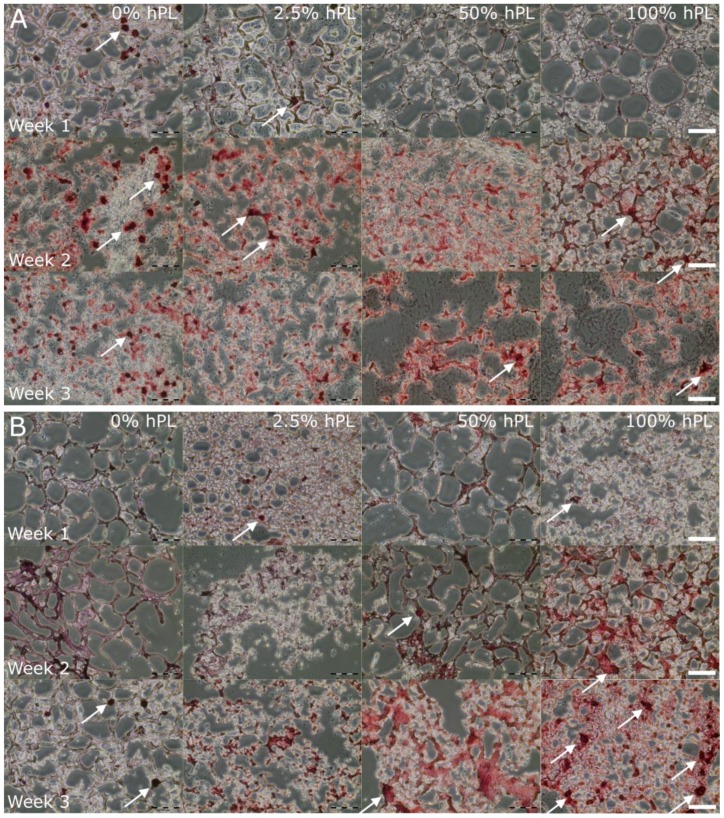
Osteogenic differentiation of AD-MSCs encapsulated in 5% (w/v) GelMA with (**A**) 50% DoF and (**B**) 70% DoF, formulated with 0, 2.5, 50, and 100% hPL. The GelMA-hydrogels were cryosectioned (12 µm) and stained with Alizarin Red 7, 14, and 21 days after induction of the osteogenic differentiation; 10× objective, scale bar 100 µm.

In order to investigate the influence of hPL on the differentiation grade of AD-MSCs in different GelMA hydrogels (50% and 70% DoF), cells were cultivated for 7, 14, and 21 days after induction of osteogenic differentiation. As illustrated in Figure 3, osteogenic differentiation of AD-MSCs was detected by Alizarin Red staining. 

**Figure A2:**
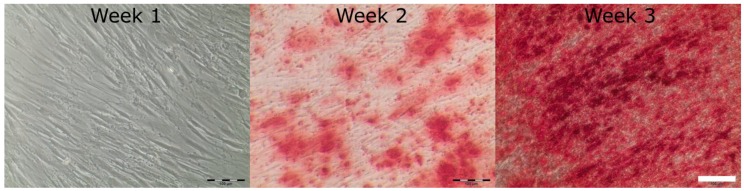
Osteogenic differentiation of 2D-cultivated AD-MSCs. The cells were stained with Alizarin Red 7, 14, and 21 days after induction of the osteogenic differentiation; 10× objective, scale bar 100 µm.

In contrast to 2D culture (Figure A2, the AD-MSCs’ cultivation in 3D constructs led to an accumulation of calcium already after one week of cultivation. Osteogenic differentiation in 50% functionalized GelMA was more pronounced with 0% and 2.5% hPL after one week. However, the addition of 50% and 100% hPL to GelMA showed a more homogeneous and intensive Alizarin Red staining in Week 3 (Figure 3A). Furthermore, GelMA with a DoF of 70% showed an increase of differentiation in Weeks 2 and 3 in GelMA hydrogels containing 50% and 100% hPL (Figure 3B). Thus, higher hPL concentrations in hydrogels increased osteogenic differentiation of AD-MSCs. In less functionalized hydrogels, bone nodules were detected earlier in GelMA hydrogels formulated with lower hPL concentrations, while higher functionalized GelMA showed increased formation of bone nodules with higher hPL concentrations. Bone nodules are formed in vivo by progenitor cells, which differentiate into osteoblasts and are thus characteristic for osteogenic differentiation [36]. A positive effect of 3D cell cultivation on the osteogenic differentiation of MSCs was also demonstrated by Re et al. in gelatin-chitosan hydrogels [9]. Our results also support the observation that 3D cultivation enhances the osteogenic differentiation of AD-MSCs and illustrates the differentiation-supporting effect of hPL.

### 3.3. Influence of hPL on GelMA Properties

The mechanical properties of hydrogels play an important role in cell fate and in the intended application of these hydrogels as, e.g., bioinks for bioprinting [37]. As shown in this study, the addition of hPL had a positive effect on spreading, proliferation, and osteogenic differentiation of AD-MSCs. As a next step, the influence of hPL on the mechanical properties of GelMA was investigated by measuring the viscosity and storage modulus in rheological experiments. 

**Figure 4:**
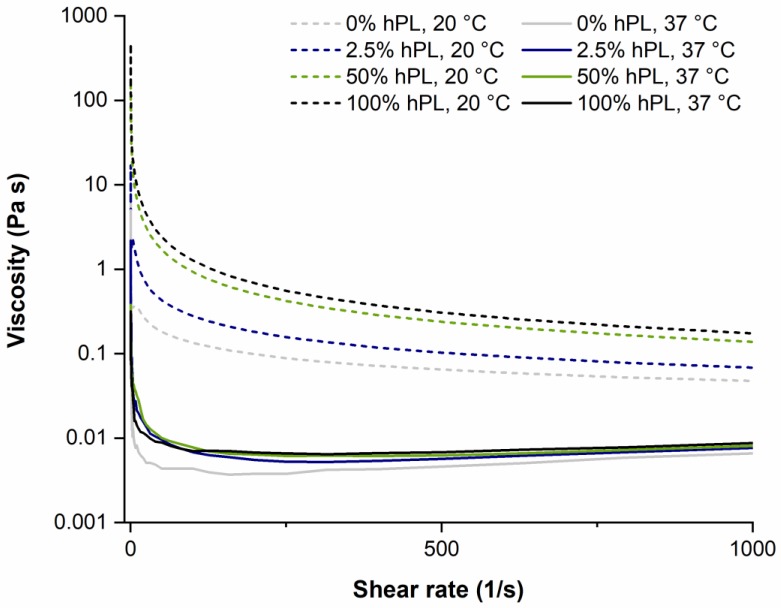
The viscosity of GelMA (5% w/v, DoF of 50%) containing 0%, 2.5%, 50%, and 100% hPL at 20 °C and 37 °C.

The viscosity of the hydrogel solution is crucial for the application in extrusion-based bioprinting. On the one hand, the solution in the cartridge syringe requires a high viscosity so that cells do not precipitate before printing. High viscosity is also desired after printing in order to maintain the printed shape before polymerization. On the other hand, a low viscosity is desirable at high pressure during extrusion (shear thinning behavior) [38]. The viscosity of GelMA solutions (DoF 50%) with different concentrations of hPL was determined by varying the shear rate from 0.1–1000 s^−1^, as these shear forces typically occur during bioprinting. The viscosity was measured at 20 and 37 °C, which corresponds to room temperature (e.g., during bioprinting) and the physiological cultivation temperature. As illustrated in Figure 4, all samples showed decreasing viscosity with increasing shear rate. Thus, GelMA showed a shear thinning flow behavior independent of hPL concentration. Furthermore, as expected, the GelMA viscosity at 20 °C was higher than at 37 °C. More interestingly, the addition of hPL increased GelMA viscosity in a concentration-dependent manner. This effect was comparable to the addition of rheological additives such as hyaluronic acid, which increase the viscosity of hydrogels [8]. These results demonstrate that the viscosity of GelMA hydrogels used as bioinks can be adjusted by varying the concentration of hPL and the temperature used for bioprinting.

**Figure 5:**
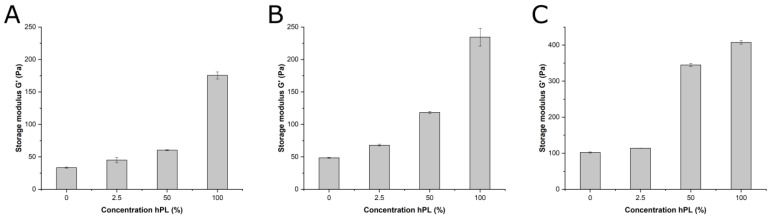
(**A**) Material stiffness of GelMA hydrogels (5% w/v) with a DoF of 50% (A) and a DoF of 70% (**B**) at 25 °C determined by a time-sweep oscillatory test. (**C**) Material stiffness of unmodified gelatin at 25 °C, determined by a time-sweep oscillatory test. Data represent the mean ± SD for a threefold determination.

The mechanical properties of hydrogels formulated with different hPL levels were investigated using rheology. As can be seen in Figure 5A–C, the storage modulus of GelMA increased with higher hPL concentrations. This effect was also observed for the parent molecule, parent gelatin material without methacrylic modification, formulated with different hPL concentrations and measured at 25 °C, where gelatin formed a physical gel (Figure 5C). At this temperature, gelatin formed a stronger gel than the modified GelMA, whose vinyl side chains led to steric hindrance [39]. Thus, hPL addition enhanced the storage modulus of a hydrogel regardless of the type of cross-linking involved (covalent photo-cross-linking or physical interactions). Apparently, the presence of additional polypeptides and macromolecules in hPL contributes to the overall viscoelastic strength of the polymer network. Similar effects were observed for rheological additives based on polysaccharides or proteins in bioinks, as well as on the presence of protein additives like whey protein on gelatin gels [40,41]. Moreover, during the time-sweep studies of GelMA polymerization, we could also observe that higher levels of hPL led to quicker onset of polymerization.

**Figure 6:**
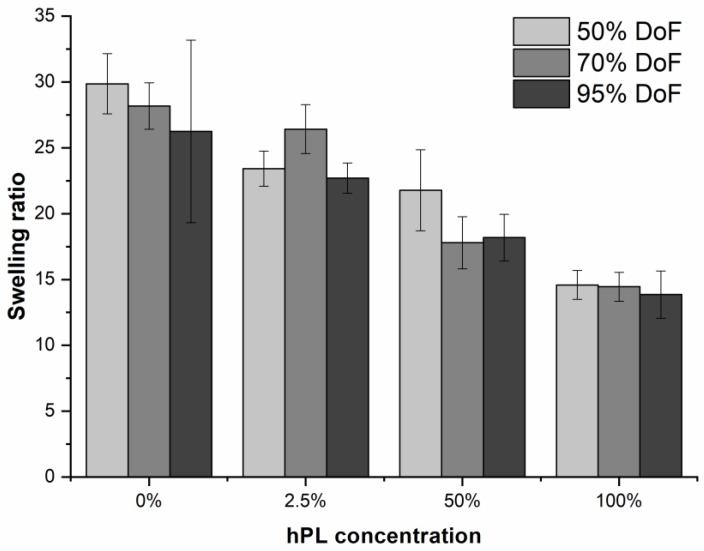
Swelling ratio of GelMA hydrogels with 50%, 70%, and 95% functionalization and the addition of 0%, 2.5%, 50%, and 100% hPL. Data represent the mean ± SD for a sixfold determination.

Hydrogels can maintain more than 90% water in their 3D network. The extent of water uptake influences the mechanical properties of a hydrogel such as overall elasticity, compressibility, and, thus, ultimately, cell behavior [12,42,43]. For this reason, it was important to investigate the influence of hPL on the swelling ratio of GelMA hydrogels. As can be seen in Figure 6, the swelling ratio decreased with increasing hPL concentration in all tested hydrogels. Incorporating hPL in the GelMA hydrogel decreased overall water uptake by the network. A lower swelling ratio typically indicates a more compact network structure with smaller pores, for example resulting due to the formation of additional cross-links. While hPL did not directly introduce more covalent cross-links into the GelMA hydrogel, the presence of hPL macromolecules reduced available interstitial volume. Another beneficial effect of a lower swelling ratio is improved maintenance of shape fidelity after bioprinting.

Taken together, these results show that the addition of hPL to GelMA not only contributed to the biological effects on the cells, but also influenced the resulting material properties of the hydrogel.

## 4. Discussion

In this study, the influence of hPL on the cell behavior of 3D cell cultures of AD-MSCs in GelMA hydrogels was investigated. In addition, the material properties of the resulting GelMA hydrogels were studied, as well. Since hPL had already shown a positive effect on the growth of different types of primary and stem cells in 2D [18,21,22,23,25,26,29,31,44,45], we hypothesized that the addition of hPL during hydrogel formulation would be beneficial for cells growing in 3D cultures. Moreover, the influence of hPL on GelMA material properties in terms of viscosity (before polymerization), storage modulus (after polymerization) and swelling ratio (after polymerization) was also previously unknown. Cultivation of MSCs in 3D seems to be advantageous, since the quality of cells (in terms of production of angiogenic and immunomodulatory factors) from 3D cultures is higher compared to 2D-expanded cells [46,47,48,49,50,51]. Moreover, physiological cell-cell and cell-matrix interactions can only be recreated in 3D cell cultures [49,52,53]. Earlier, we demonstrated that GelMA hydrogels of different DoFs can be tuned to change their mechanical properties (e.g., UV intensity and hydrogel concentration), which in turn influences MSC growth and behavior [10].

Traditionally, PBS or other buffer solutions are used to formulate or reconstitute hydrogels [8,32,54,55,56,57,58]. In our work, we substituted PBS partially or fully with hPL. The addition of hPL led to a concentration-dependent increase in cell viability and osteogenic differentiation. When cells were encapsulated in hydrogels formulated with PBS only, important nutrients and adhesion factors, which are missing in the bulk hydrogel, must diffuse from the cultivation media or be gradually produced by the cells themselves. Kaemmerer et al. measured the diffusion rate of a 70-kDa molecule in GelMA of DoF 70% [39]. The authors could show that the diffusion of the test molecule inside the hydrogel construct was 23.4 µm^2^/s and that it was slower than diffusion in water alone. hPL contains molecules of a great variety of sizes (e.g., fibronectin = 440 kDa), so the exact diffusion rate of all proteins cannot be determined. In addition, the phase boundary between gel and liquid medium represents an additional diffusion barrier. As a natural derivative from collagen I, GelMA provides sufficient arginine-glycine-aspartic acid (RGD) sequences for cell adhesion [59,60,61,62,63]. The presence of natural RGD sequences in GelMA leads to cell adhesion and spreading of encapsulated cells typically after three days of cultivation, as shown by our group [10] and other researchers [12]. In contrast, adhesion to the plastic cell culture plate occurred after 24 h in 2D. In situ formulation of hydrogels with hPL leads to cell attachment and spreading only after one day of cultivation in 3D. One possible explanation for this observation is the presence of vitronectin and fibronectin in hPL. Indeed, the α-granules of platelets contain different types of adhesion proteins, including von Willebrand factor (vWF), fibronectin, vitronectin, and thrombospondin [35,64,65,66]. The exact reasons for the positive hPL effects on cell adhesion and spreading in hydrogels must be investigated in greater detail in future studies. Fast and effective cell adhesion and spreading are extremely important for anchorage-dependent cells like MSCs [67], since adhesion and spreading regulate their proliferation, migration, and differentiation [67,68,69]. 

Thus, the supplementation of hydrogels with hPL directly during encapsulation and polymerization can positively influence the cells via both (I) the provision of growth and differentiation factors and (II) the enhancement of cell spreading and adhesion in the hydrogel. This is essential for fast ex vivo tissue formation. Moreover, both autologous and allogenic hPL can be used for hydrogel supplementation, which creates the possibility of individualized off-the-shelf TE constructs. The positive effect of hPL addition to the cell culture medium during cultivation of 3D cell cultures was also recently supported by the studies of Re et al. [9]. This group also found that the addition of 5% hPL to the medium increased the proliferation and osteogenic differentiation of bone marrow (BM)- and AD-MSCs encapsulated in gelatin-chitosan hybrid hydrogels [9]. Only three different hPL concentrations (2.5%, 50%, and 100%) were investigated to study the general outcome of PBS substitution during GelMA formulation. Further experiments, using hPL concentrations at levels between tested ones, could lead to even better protocols. Moreover, the influence of hPL addition on other cell types must be studied as well. In future experiments, the combination of the use of hPL for hydrogel formulation and for cell culture medium supplementation during expansion (2D) and subsequent cultivation in 3D must be also studied, since such a combined protocol could further enhance cell growth and differentiation. In our work, we demonstrated for the first time that the addition of hPL directly to the hydrogels during formulation and polymerization displayed positive effects on AD-MSCs.

Cell behavior and especially cell fate are directly related to the stiffness of matrix materials [70,71,72,73,74,75]. Thus, soft tissue supports the differentiation of stem cells into neural lineages, while stiffer materials support the differentiation into muscle cells. Finally, hard and rigid materials lead to osteogenic differentiation [70,71,72,73,74,75]. In this study, three GelMA materials with different degrees of functionalization and thus, stiffness, were used to study the effect on cell growth. A material of 50% DoF was compared to a material of higher mechanical strength (70% DoF). Indeed, we could show that a higher degree of osteogenic differentiation was achieved at the higher DoF and thus at higher mechanical strengths. Moreover, the addition of hPL was shown to contribute to an increase in the overall mechanical strength of the material, which also promoted osteogenic differentiation.

3D bioprinting represents an advanced 3D cell culture technique, where higher spatial control of cell distribution/organization can be achieved. Earlier, we reported that GelMA hydrogels can be used for bioprinting, but that such bioinks would require viscosity enhancers for printing at the low GelMA concentrations supporting AD-MSCs cell spreading [10]. The results of the current work demonstrate that supplementation with hPL alone increases hydrogel viscosity at room temperature, which in turn makes hPL an interesting addition to the bioink toolkit. However, even higher viscosities are usually required for high fidelity extrusion bioprinting [76]. In our lab, we typically use viscosities between 20 and 200 mPa·s for the bioprinting of cell-promoting bioinks. This viscosity range, however, will necessitate the addition of rheological additives or even lower printing temperatures during bioprinting of hPL-formulated GelMA. As an additional effect, hPL decreases the swelling ratio of the hydrogel, which is beneficial for maintaining shape fidelity after printing. Of course, the true printability of GelMA hydrogels formulated with hPL has to be evaluated in future studies. 

Taken together, our results demonstrated that the supplementation of GelMA hydrogels with different degrees of functionalization with hPL had a positive effect on cell spreading, proliferation, and differentiation. Moreover, the viscosity of GelMA solutions and the storage modulus of cross-linked hydrogels were increased by the addition of hPL. These results can be used to improve the protocols/methods for hydrogel-based tissue engineering and for extrusion-based bioprinting.
